# Leptin Receptor Expression in Mouse Intracranial Perivascular Cells

**DOI:** 10.3389/fnana.2018.00004

**Published:** 2018-01-23

**Authors:** Xuefeng Yuan, Alexandre Caron, Hua Wu, Laurent Gautron

**Affiliations:** ^1^Division of Hypothalamic Research and Department of Internal Medicine, The University of Texas Southwestern Medical Center, Dallas, TX, United States; ^2^Department of Orthopedics, Tongji Hospital, Tongji Medical College, Huazhong University of Science and Technology, Wuhan, China

**Keywords:** adipokines, neuroanatomy, pericytes, signaling, transgenic mice

## Abstract

Past studies have suggested that non-neuronal brain cells express the leptin receptor. However, the identity and distribution of these leptin receptor-expressing non-neuronal brain cells remain debated. This study assessed the distribution of the long form of the leptin receptor (LepRb) in non-neuronal brain cells using a reporter mouse model in which LepRb-expressing cells are permanently marked by tdTomato fluorescent protein (LepRb-Cre^tdTomato^). Double immunohistochemistry revealed that, in agreement with the literature, the vast majority of tdTomato-tagged cells across the mouse brain were neurons (i.e., based on immunoreactivity for NeuN). Non-neuronal structures also contained tdTomato-positive cells, including the choroid plexus and the perivascular space of the meninges and, to a lesser extent, the brain. Based on morphological criteria and immunohistochemistry, perivascular cells were deduced to be mainly pericytes. Notably, tdTomato-positive cells were immunoreactive for vitronectin and platelet derived growth factor receptor beta (PDGFBR). *In situ* hybridization studies confirmed that most tdTomato-tagged perivascular cells were enriched in leptin receptor mRNA (all isoforms). Using qPCR studies, we confirmed that the mouse meninges were enriched in *Leprb* and, to a greater extent, the short isoforms of the leptin receptor. Interestingly, qPCR studies further demonstrated significantly altered expression for *Vtn* and *Pdgfrb* in the meninges and hypothalamus of LepRb-deficient mice. Collectively, our data demonstrate that the only intracranial non-neuronal cells that express LepRb in the adult mouse are cells that form the blood-brain barrier, including, most notably, meningeal perivascular cells. Our data suggest that pericytic leptin signaling plays a role in the integrity of the intracranial perivascular space and, consequently, may provide a link between obesity and numerous brain diseases.

## Introduction

Leptin’s metabolic actions are mediated by its binding to the long-form of the leptin receptor (LepRb) within specific brain regions (Coppari et al., 2005; de Luca et al., 2005; Leinninger et al., 2009; Fernandes et al., 2015). LepRb has a cytoplasmic tail responsible for the phosphorylation and activation of signal transducer and activator for transcription 3 (pSTAT3) (Vaisse et al., 1996; Bjorbaek et al., 1997). Numerous studies have investigated the distribution of LepRb-expressing cells in the rodent brain by using neuroanatomical techniques such as *in situ* hybridization and immunohistochemistry for pSTAT3 (Hakansson et al., 1996; Elmquist et al., 1997; Mercer et al., 1998; Hubschle et al., 2001; Munzberg et al., 2003; Fulton et al., 2006; Caron et al., 2010; Laque et al., 2013; Maniscalco and Rinaman, 2014; Lima et al., 2016). Transgenic mouse lines that express reporter proteins under the control of the LepRb gene promoter have also been used to map LepRb-expressing brain cells (Leshan et al., 2009, 2010; Scott et al., 2009; Patterson et al., 2011; Lima et al., 2016).

Together, the above studies have unequivocally demonstrated that the vast majority of leptin-responsive cells in the adult rodent brain are neurons. However, parallel studies also reported that non-neuronal brain cells may be sensitive to leptin. In particular, leptin binding and mRNA for the short isoforms of the leptin receptor were both detected in the choroid plexus, meninges, astrocytes and the brain vasculature (Devos et al., 1996; Guan et al., 1997; Bjorbaek et al., 1998; Boado et al., 1998; Corp et al., 1998; Elmquist et al., 1998; Hileman et al., 2002; Carlo et al., 2007; Koga et al., 2014). The short isoforms of the leptin receptor have been implicated in the transport of leptin across the blood-brain barrier (Thomas et al., 2001; Hsuchou et al., 2013; Li et al., 2013). *Leprb* mRNA and leptin-induced pSTAT3 have also been reported in the brain vasculature, choroid plexus, 3rd ventricle, and the median eminence (Mutze et al., 2006; Frontini et al., 2008; Cottrell et al., 2009; Balland et al., 2014; Rottkamp et al., 2015). Furthermore, *Leprb* mRNA and immunoreactivity have been detected in astrocytes (Hsuchou et al., 2009a,b; Bosier et al., 2013; Kim et al., 2014). Enrichment of vascular markers was also recently reported in LepRb-expressing brain cells (Allison et al., 2015). *In vitro*, both microglial cells and astrocytes were shown to be responsive to leptin pre-treatment (Pinteaux et al., 2007; Lafrance et al., 2010; Fuente-Martin et al., 2012).

Despite the aforementioned evidence, expression of LepRb in non-neuronal cells, including astrocytes, remains controversial for several reasons: (1) the administration of leptin only induces pSTAT3 in neurons but not in adult glial-fibrillary protein acidic (GFAP)-positive brain cells (Mutze et al., 2006; Caron et al., 2010; Rottkamp et al., 2015); (2) fluorescently tagged LepRb-expressing cells in transgenic reporter mouse models do not resemble non-neuronal cells (Leshan et al., 2009, 2010; Scott et al., 2009; Patterson et al., 2011); (3) the specificity of commercially available antibodies against the LepRb protein has been questioned by investigators in the field (Scott et al., 2009; Kim et al., 2014); (4) transcriptomic databases of human and mouse astrocytes do not reveal significant LepRb enrichment when compared to other cell types (Zhang et al., 2014, 2016); (5) LepRb is enriched in mouse arcuate nucleus neurons but not in glial cells (Campbell et al., 2017); and 6) likewise, LepRb-expressing cells from the entire mouse brain are not enriched in glial markers (Allison et al., 2015). With these inconsistencies in mind, the present study sought to reassess the distribution of LepRb-driven Cre recombinase activity in non-neuronal cells of the mouse brain. Collectively, our data indicate that the only non-neuronal cells with significant leptin receptor expression in the adult mouse brain are cells that make up the blood-brain barrier, including, most notably, meningeal perivascular cells.

## Materials and Methods

### Animals

LepRb-Cre mice originally generated in the Friedman Laboratory (DeFalco et al., 2001) were maintained and genotyped as previously described (Scott et al., 2009). Mice were crossed with tdTomato reporter mice from the Jackson Laboratory (stock#007905) (Madisen et al., 2010; Gautron et al., 2011). Unpublished experiments in our laboratory have shown that the distribution and intensity of tdTomato cells is identical in mice carrying one or two Cre and/or tdTomato alleles. Here, we used LepRb-Cre^tdTomato^ mice carrying one Cre and one floxed-tdTomato allele. A total of 20 adult male mice (4–12 weeks old) were used to assess the distribution of tdTomato across the brain (*n* = 3), double immunohistochemistry (*n* = 5), morphological studies (*n* = 3), dual *in situ* hybridization (*n* = 3), and the presence of pSTAT3 (*n* = 6). All of the above mice were on a pure C57Bl6/J genetic background.

C57BL/6 mice were also purchased from the UT Southwestern Animal Resource Center. Adult male mice (6–8 weeks old) were used to validate our brown *in situ* hybridization (*n* = 3). Adult male LeprloxTB mice (Berglund et al., 2012) in which transcriptional blocking sequences effectively block transcription of LepRb (referred to as LepRb-deficient mice), and their littermates, were used for qPCR experiments (*n* = 4/genotype).

All of our mice were housed with *ad libitum* access to both food and water in a light- (12 h on, 12 h off) and temperature-controlled (21.5–22.5°C) barrier facility. Animal work described in this manuscript has been approved and conducted under the oversight of the UT Southwestern Institutional Animal Care and Use Committees (APN# 1090-06-02-1 and 2017-101-994).

### Leptin Treatment

Recombinant leptin from mouse (Sigma–Aldrich, cat# L3772) diluted in sterile saline was injected intraperitoneally (i.p.) to LepRb-Cre^tdTomato^ mice at a dose of 4 mg/kg. Control animals received 300 ml of sterile saline. For pSTAT3 studies, mice were sacrificed at 45 min post-injection. The dosage, time points, and route of administration were chosen based on prior literature (Caron et al., 2010; Bouret et al., 2012; Gautron et al., 2013).

### qPCR

Total mRNA was isolated from the mediobasal hypothalamus (H) and meninges (M) using STAT60 reagent (Amsbio, Cambridge, MA, United States). The RNA concentrations were estimated from absorbance at 260 nm. cDNA synthesis was performed using High Capacity cDNA Kit (Applied Biosystems). Extraction of mRNA and cDNA synthesis were performed following the manufacturer’s instructions. cDNA was diluted in DNase-free water before quantification by real-time PCR. Transcript levels were measured in duplicate samples using a ABI 7900 HT Sequence Detection System (Applied Biosystems). The relative expression of target mRNAs was calculated using the 2Δ-cycle threshold method by comparison with the housekeeping gene beta-2-microglobulin (B2m). TaqMan probes used in this study are shown in **Table 1**. Data are expressed as mean ± SEM. Comparison between littermates and LeprloxTB mice were analyzed by Student’s unpaired *t*-test. All statistical tests were performed using GraphPad Prism (version 7.0), and *p* < 0.05 was considered statistically significant.

**Table 1:** List of reagents used for immunohistochemistry, *in situ* hybridization, and qPCR.

Antigen	Manufacturer	Cat#	Dilution	Labels
**Primary antibodies**				
NeuN	Cell signaling	12943	1:1,000	Alexa Fluor 488
DsRed	Clontech	632496	1:1,000	Alexa Fluor 488
Iba1	Wako	019-19741	1:1,000	Alexa Fluor 488
pSTAT3	Cell Signaling	9131	1:2,000	DAB-Nickel-Cobalt
GFAP	Sigma–Aldrich	G9269	1:1,000	Alexa Fluor 488
Vitronectin	GenWayBio	GWB-794F8F	1:500	Alexa Fluor 488
PDGFRB	R&D	AF1042	1:150	Alexa Fluor 488

**Gene name**	**Accession #**	**Target region**	**Cat#-channel**	**Chromogenic labels**

**ISH RNAScope probes from ACD**				
DapB	EF191515	414–862	310043-c1	DAB–brown
Ppib	NM_011149.2	98–856	313911-c1	DAB–brown
LepR	U42467.1	1361–2317	402731-c1	DAB–brown or FastRED–red or Blue
Gfap	NM_001131020.1	2–1761	313211-C2	Red
Pdgfrb	NM_001146268.1	3083–4090	411381-C2	Red
Aif1	NM_019467.2	31–866	319141-C2	Red

**Gene name**	**Accession #**			

**qPCR probes**				
Lepr (all)	Mm00440181_m1			
Lepr (a)	Mm01262070_m1			
Lepr (b)	Mm01265583_m1			
Vtn	Mm00495976_m1			
Pdgfrb	Mm00435546_m1			
Acta1	Mm00808218_g1			
Cspg4	Mm00507257_m1			
Rbfox3	Mm01248771_m1			
Pomc	Mm0435874_m1			
Npy	Mm00445771_m1			
B2m	Mm00437762_m1			

### Tissue Preparation

Mice were anesthetized with chloral hydrate (500 mg/kg, i.p.) and perfused transcardially with 0.9% saline followed by 10% neutral buffered formalin (Sigma–Aldrich) for 2 min. Brains and dura mater were dissected and post-fixed in formalin for 1 h, unless otherwise indicated. Next, samples were incubated overnight at 4°C in 20% sucrose made in phosphate-buffered saline (PBS), pH 7.0. For immunohistochemistry, brains were sectioned at 25 μm thickness using a freezing microtome. Brain coronal sections were collected in PBS and stored in cryoprotectant at -20°C. For *in situ* hybridization studies, 14 μm brain coronal sections were collected on SuperFrost slides using a cryostat, and kept at -80°C. For imaging native tdTomato in whole mounts of the dura mater, samples were not incubated in sucrose, but instead processed for immunohistochemistry and/or mounted on slides, and a coverslip with Vectashield containing DAPI was placed on the samples (Vectastain).

### Immunofluorescence Studies

Free-floating brain sections were incubated overnight at room temperature in a blocking solution (0.1% Triton X-100, 2% normal serum in PBS) containing the primary antisera for NeuN, Gfap, Iba1, vitronectin and PDGFRB (see **Table 1**). On the following day, sections were incubated with a biotinylated donkey anti-rabbit or anti-goat antibody (Jackson ImmunoResearch #711-066-152 or 705-065-147), followed by Streptavidin Alexa Fluor 488 (1:1,000; Invitrogen).

Biotinylated isolectin B4 (IsB4) from Griffonia simplicifolia (cat#L2140) binding was visualized by incubating the slides with a solution containing 8 μg/ml IsB4 in a Tris NaCl buffer with 0.1 mM CaCl_2_ and 0.1 mM MgCl_2_. After several washes, sections were incubated with biotinylated secondary antibody (1:1,000 donkey anti rat; Jackson Immunoresearch, United States) and then with Streptavidin Alexa 488 (1:1,000; Invitrogen). Finally, a coverslip with Vectashield containing DAPI was placed on the samples (Vectastain).

### pSTAT3 Immunohistochemistry

We performed pSTAT3 staining on fixed frozen free-floating brain sections. Sections were pretreated with solutions containing NaOH, hydrogen peroxide, glycine, and sodium dodecyl sulfate, as previously described (Gautron et al., 2013). Then, sections were incubated overnight at room temperature in a rabbit polyclonal antiserum raised against pSTAT3 (see **Table 1**). On the following day, sections were incubated with a biotinylated donkey anti-rabbit secondary antibody, followed by a solution of Vectastain ABC elite protocol (Vector Laboratories) and 3,3-diaminobenzidine (DAB) mixed with Nickel-Cobalt (5% Cobalt Chloride and 1% Nickel Sulfate). In our hands, tdTomato native fluorescence was slightly attenuated following pSTAT3 immunohistochemistry.

### *In Situ* Hybridization

#### RNAScope Chromogenic Assay for Fresh Frozen Samples

As previously described by us (Liu et al., 2015), C57/Bl6 mice received an overdose of chloral hydrate (500 mg/kg, i.p.) and their brains were rapidly dissected and frozen on a bed of dry ice. Using a cryostat, 14 μm brain sections (1:5 series) were collected on SuperFrost slides and stored at -80°C. Following the manufacturer’s protocol (Advanced Cell Diagnostic), brain sections were hybridized with double-Z oligo probes for *Lepr* (all isoforms), *Ppib* (positive control) and *Dapb* (negative control) (**Table 1**). Signal detection was achieved using DAB and tissue was counterstained with hematoxylin. Finally, coverslips with EcoMount were placed on the samples (FisherScientific).

#### RNAScope Chromogenic Assay Combined with Immunohistochemistry for Fixed Frozen Samples

LepRb-Cre^tdTomato^ adult male mice were perfused as previously described. Fixed brains were kept in 10% formalin for an additional 48–72 h at 4°C. After post-fixation, brains were transferred into a solution of 20% sucrose in PBS for 24 h, before being frozen on a bed of dry ice and stored at -80°C. Brain sections with a thickness of 14 μm were placed onto SuperFrost Plus slides (1:5 series). On the hybridization day, the tissue was pretreated following a protocol modified from the ACD Preparation Technical Note for Fixed Frozen Tissue. Briefly, slides were baked at 60°C for 30 min, washed in 1X PBS, and then treated in H_2_O_2_ for 10 min. Pretreatment consisted of hot 1X Target Retrieval solution for 1–2 min. Next, slides were rinsed in distilled water and dehydrated in fresh 100% ethanol. Tissue was incubated with Pretreatment 3 at 40°C for 15 min. After rinsing the slides in distilled water, hybridization was performed using the standard ACD procedure and reagents from the RNAscope^®^ 2.5 HD Detection Kit (RED). The LepR-C1 probe (**Table 1**) was prepared and applied to our tissue at 40°C for 2 h. Amplification steps were carried out following the manufacturer’s instructions. Lastly, signal detection was achieved using a mix of Fast RED-B and Fast RED-A in a ratio of 1:60 at room temperature for about 10 min. Slides were washed in distilled water. Native tdTomato fluorescence was greatly diminished following *in situ* hybridization. Therefore, we labeled brain sections using a DsRed rabbit antibody that detects tdTomato. EcoMount mounting medium was applied over the sections. Pilot experiments in adjacent tissue sections were also performed to ensure that *in situ* hybridization and immunohistochemistry minimally altered the efficacy of each individual procedure.

### Microscopy

#### Bright-Field and Epifluorescence

Images of DAB-labeled tissues were captured using the standard fluorescent and bright-field optics of a Zeiss Axioplan light microscope attached to a digital camera. This microscope was also used for counting the number of tdTomato-positive cells per brain section.

#### Confocal

Digital images were acquired using the 63x oil objective of Leica Sp5 confocal laser scanning microscopes (UT Southwestern Live Imaging Core Facility). We collected Z-stacks of images throughout the area of interest (3–12 planes with an increment varying 0.35–1 μm and a digital zoom up to 3) at a resolution of 512 × 512 pixels, and a line average of either 8 or 16. Projection of the Z-stacks to a single image was performed in ImageJ. Furthermore, ImageJ was used to generate plot profiles for confocal images in each channel of interest by tracing a line between two chosen pixels. The measuring tool calculated the averaged pixel values along this arbitrary line.

#### Estimates

We manually counted tdTomato fluorescent cells and select immuno-labeled proteins. Cell types including meningeal cells, perivascular cells, neurons and epithelial cells were differentiated based on their location and shapes. Cell counts were performed using a 20x objective and data are presented as the total number of cells per coronal brain section across identified brain areas. The anatomical nomenclature in this study is taken from the Franklin and Paxinos Mouse Brain Stereotaxic Atlas (3rd edition). Our counts are only meant to provide relative data but not accurate counts of absolute cell numbers. We also provided estimates of *Lepr* mRNA- and tdTomato-positive cells. Colocalization was subjectively determined by visual inspection of coronal brain sections. The RNAscope approach generated virtually no background or unspecific labeling, thereby facilitating the identification of mRNA-expressing cells. Individual profiles containing as little as one red dot were considered positive for *Lepr* mRNA.

#### Illustrations

We used Adobe Photoshop CS5 to make the following changes to our digital images including: (1) Uniformly adjusting of the resolution (300 dpi), cropping, contrast, and highlights; (2) Converting DAPI to gray using the Desaturate tool; (3) Merging images from different channels using either the Lighten or Screen overlay features; (4) Generating annotations and scale bars; (5) Assembling fluorescence plot profiles.

## Results

### Characterization of Intracranial LepRb-Cre Activity in the Mouse

**FIGURE 1:**
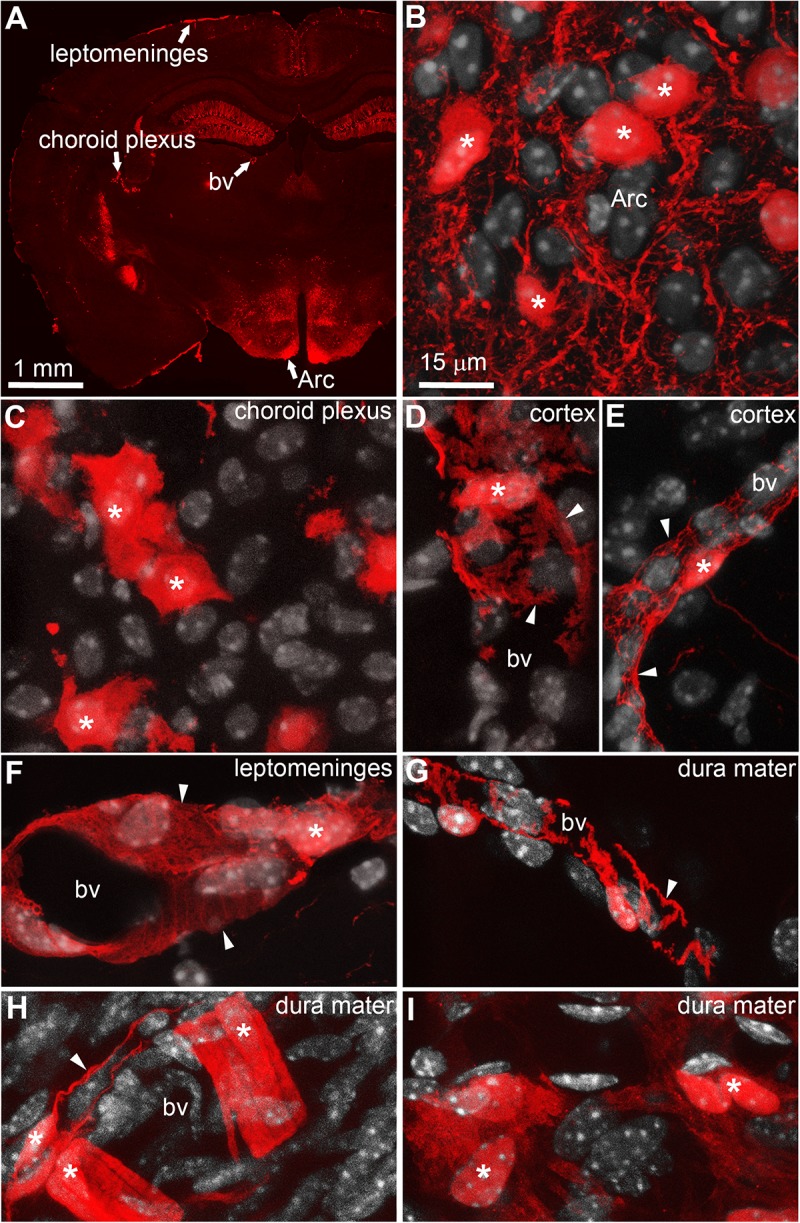
Distribution of intracranial tdTomato-labeled cells in the LepRb-Cre-tdTomato mouse. **(A)** Brain section at the level of the hypothalamus. Bright native fluorescence can be observed across the mouse brain in both the gray matter and structure outside the blood-brain barrier including the leptomeninges, and choroid plexus and isolated blood vessels. **(B)** Representative tdTomato-labeled neurons located in the arcuate nucleus of the hypothalamus (asterisks). Please note somas surrounded by a dense network of passing axons. **(C)** The choroid plexus containing clusters of tdTomato-labeled cells following the distribution and shape of epithelial cells (asterisks). **(D,E)** Representative tdTomato-labeled cells with a “bump-on-a-log” morphology in the cortex (asterisks). The latter cells are typically sparse with digitized processes wrapping around small blood vessels (arrows). **(F–H)** The leptomeninges and dura mater contained dense populations of cells in a perivascular position (asterisks). Despite the varied size and shape of their processes (arrows), most meningeal tdTomato-labeled cells in size resembled mural cells known as pericytes. **(I)** Occasionally, clusters of tdTomato-labeled cells resembling fibroblasts were seen in the dura matter. DAPI was converted to gray to enhance its visibility. Scale bar in **(B)** applies through **(I)**. Arc, arcuate nucleus; bv, blood vessel.

Native tdTomato fluorescence was observed across the brain of LepRb-Cre^tdTomato^ mice in a pattern that was identical to that which was previously reported using the same Cre line (Scott et al., 2009). Based on their shape and distribution, the majority of tdTomato-tagged cells were deduced to be neurons. For example, within the arcuate nucleus of the hypothalamus, we observed large cell bodies surrounded by a dense network of varicose processes (**Figures 1A,B**). However, fluorescence was consistently observed in regions devoid of neurons including the choroid plexus (**Figure 1C**), the perivascular space (**Figures 1D,E**), the leptomeninges (**Figure 1F**), and dura matter (**Figures 1G–I**). Within the choroid plexus epithelium, tdTomato was observed in cells with the morphology of epithelial cells (**Figure 1C**). Despite their varied morphology, tdTomato-tagged cells located in the meninges and around blood vessels displayed the typical “bump on a log” shape and processes of perivascular cells known as pericytes (**Figures 1D–G**) (Winkler et al., 2011; Hill et al., 2015; Attwell et al., 2016; Trost et al., 2016). Notably, tdTomato-tagged perivascular cells were sparse around brain parenchyma blood vessels and were often located around cortical blood vessels immediately protruding off of the leptomeninges (**Figures 1D,E**). In most brain regions, we rarely observed more than a dozen of tdTomato perivascular cells per section. In comparison, tdTomato-tagged cells were more abundant in the leptomeninges and dura matter. Fluorescent meningeal cells were found along the entire length of most blood vessels (**Figures 1F–I**). Occasionally, tdTomato-tagged cells located in the meninges displayed a shape more similar to that of smooth muscle cells (**Figure 1H**) or meningeal fibroblasts (**Figure 1I**). In the meninges, tdTomato-tagged cells were already present at birth, but in relatively low numbers (**Supplementary Figure S1**). Their numbers steadily increased throughout the first postnatal week.

**FIGURE 2:**
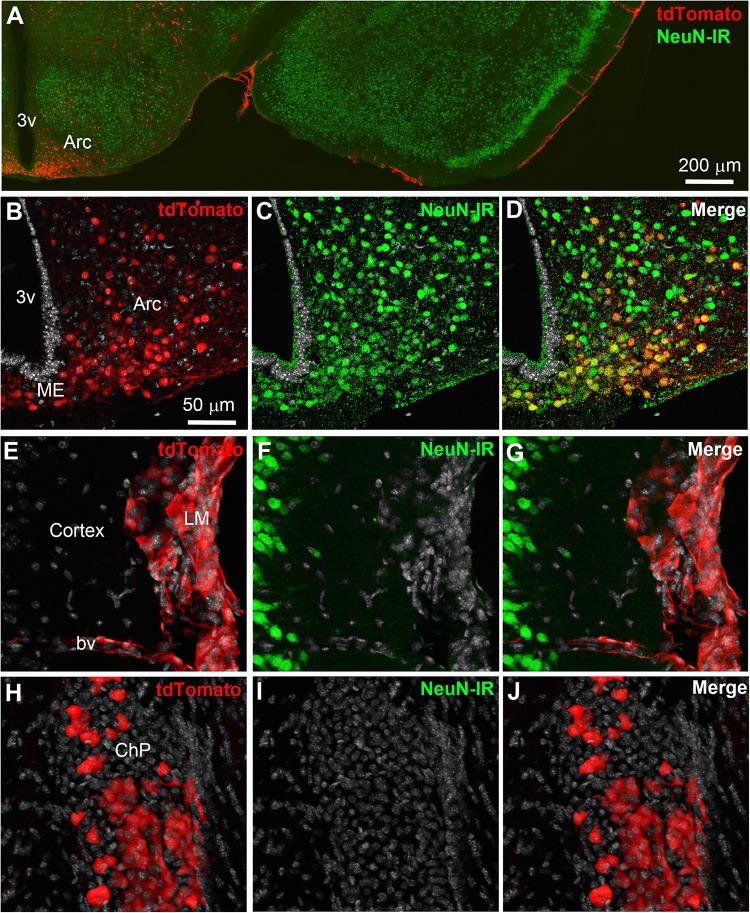
Distribution of NeuN immunoreactivity relative to tdTomato-labeled cells. **(A–D)** Distribution of nuclear NeuN immunoreactivity (green) in the mediobasal hypothalamus of LepRb-Cre-tdTomato mice. Immunoreactivity was present of all tdTomato-labeled cells in the parenchyma including the arcuate nucleus of the hypothalamus. **(E–G)** Fluorescence for tdTomato was seen in area completely devoid of neurons such as the leptomeninges and around scattered blood vessels. **(H–J)** The choroid plexus also contained many epithelial TdTomato-labeled cells. DAPI was converted to gray. Scale bar in **(B)** applies through **(J)**. Arc, arcuate nucleus; bv, blood vessel; ChP, choroid plexus; LM, leptomeninges; ME, median eminence.

**FIGURE 3:**
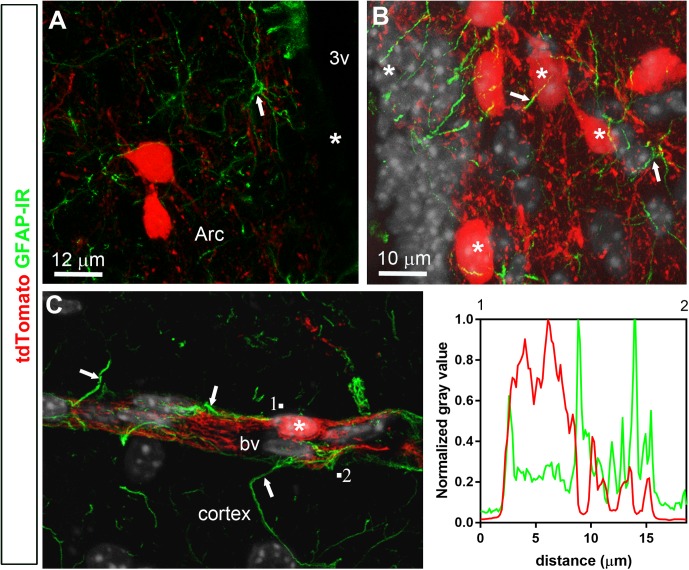
Distribution of GFAP immunoreactivity relative to tdTomato-labeled cells. **(A,B)** GFAP immunoreactivity was evident in astrocytic processes approaching very closely tdTomato cells (asterisks) in the hypothalamus and cortex, among other representative brain regions. However, GFAP never colocalized with tdTomato fluorescence and, furthermore, tdTomato-labeled cells never resembled astrocytes. **(C)** Representative tdTomato cell in a perivascular position is seen in close apposition to GFAP processes (white arrows). However, intensity plot profiles (between 1 and 2) demonstrated that GFAP did not coincide well with tdTomato. DAPI was converted to gray. Scale bar in **(B)** applies to **(C)**. Arc, arcuate nucleus; bv, blood vessel.

**FIGURE 4:**
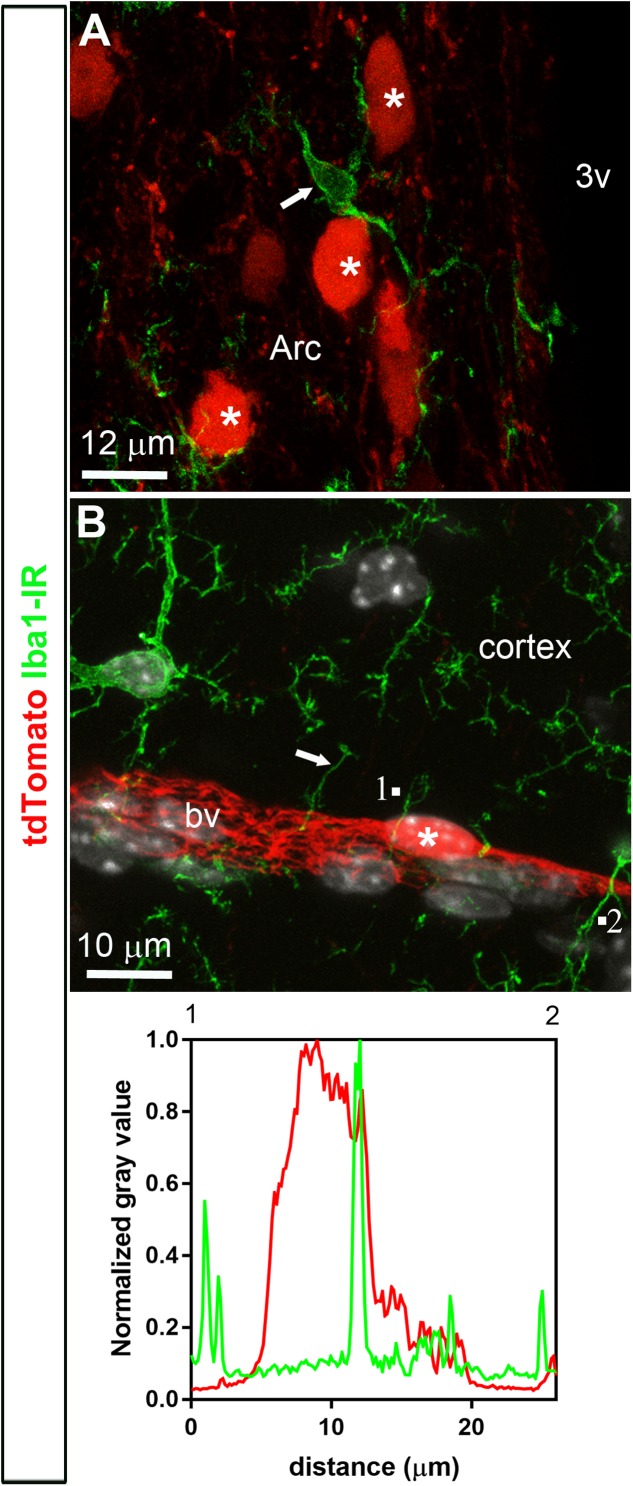
Distribution of Iba1 immunoreactivity relative to tdTomato-labeled cells. **(A,B)** Microglial cells throughout the brain were positive for Iba1 (arrows). In spite of the close proximity between microglial cells and tdTomato-labeled cells (asterisks), Iba1 never colocalized with tdTomato fluorescence and, furthermore, tdTomato-labeled cells never resembled microglial cells. The intensity plot profiles (between 1 and 2) demonstrated that Iba1 immunoreactivity did not coincide well with a tdTomato-positive perivascular cell. DAPI was converted to gray. Arc, arcuate nucleus; bv, blood vessel.

**FIGURE 5:**
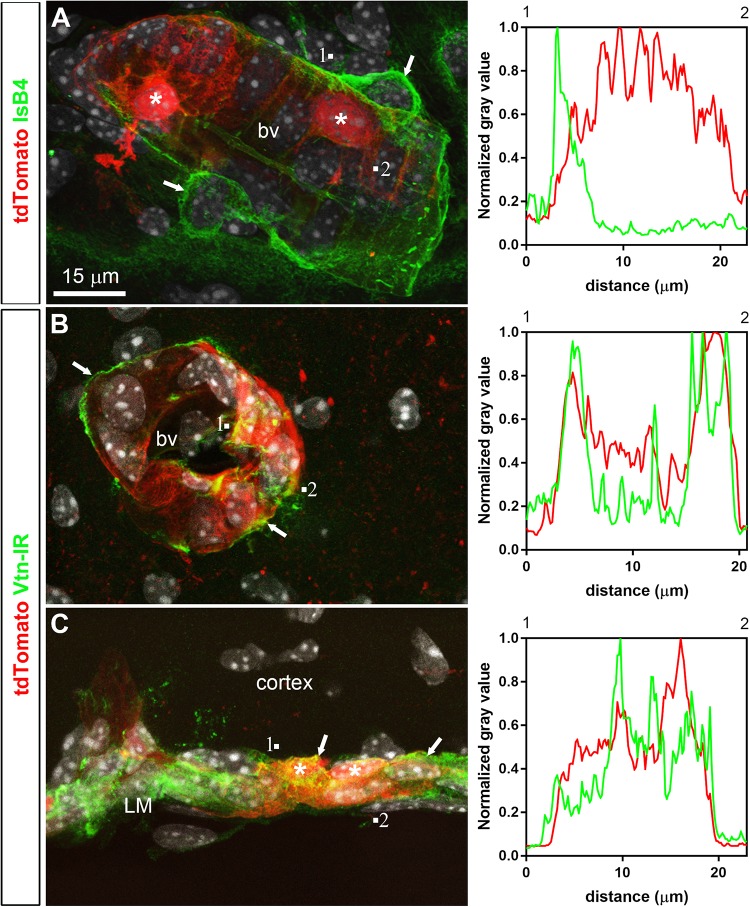
Distribution of perivascular markers relative to tdTomato-labeled cells. **(A)** Isolectin B4 binding occurred at the surface of endothelial cells, around perivascular macrophages (arrows) and perivascular tdTomato cells (asterisks). **(B,C)** Vitronectin immunoreactivity was observed decorating the abluminal surface of blood vessels in the parenchyma and leptomeninges (arrows). Intensity plot profiles (between 1 and 2) demonstrated that the surface of most perivascular tdTomato-labeled cells (asterisks) was immunoreactive for vitronectin. DAPI was converted to gray. Scale bar in **(A)** applies through **(C)**.

**FIGURE 6:**
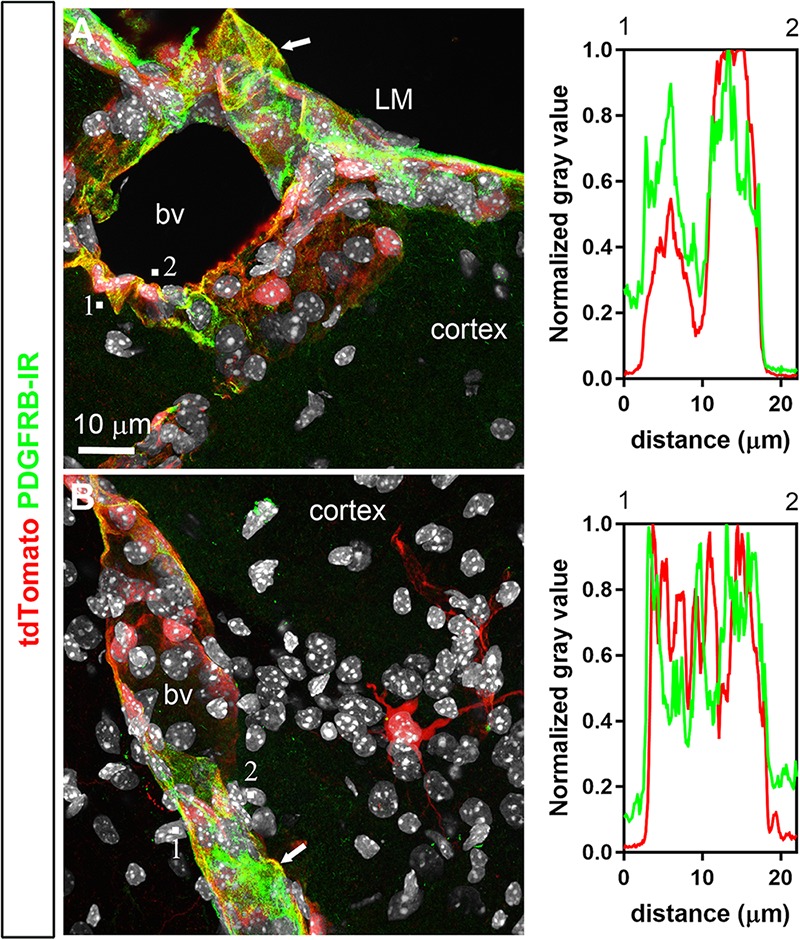
Distribution of PDGFRB immunoreactivity relative to tdTomato-labeled cells. **(A,B)** PDGFRB immunoreactivity was prominent in the leptomeninges (LM) and around parenchymal blood vessels (bv) and coincided well with the distribution of tdTomato. Most perivascular tdTomato-labeled cells were immunoreactive for PDGFRB (arrows; see also intensity plot profiles). In contrast, tdTomato neurons were not positive for PDGFRB. DAPI was converted to gray. Scale bar in **(A)** applies to **(B)**.

Double-labeled immunohistochemistry with the neuronal marker NeuN confirmed our morphological observations (**Figure 2A**). Specifically, tdTomato-tagged cells across the brain parenchyma, including those in the arcuate nucleus of the hypothalamus, were always immunoreactive for NeuN (**Figures 2B–D**). Notably, fluorescence was never seen in the lining of the 3rd ventricle (**Figures 2A,B**). TdTomato-tagged cells in the meninges, the perivascular space, and the choroid plexus were not immunoreactive for NeuN (**Figures 2E–J**). Double-labeled immunohistochemistry with prototypical glial markers including GFAP and Iba1 also confirmed the absence of tdTomato-positive glial cells (**Figures 3A–C**). Specifically, we never observed tdTomato-tagged cells in the arcuate nucleus, around blood vessels, or in any examined brain regions that unambiguously resembled glial cells or that were immunoreactive for GFAP or Iba1 (**Figures 3**, **4**). Further confirming their perivascular nature, tdTomato-tagged cells lay directly alongside isolectin b4–positive endothelial cells (**Figure 5A**). Although universal markers for pericytes are not available, recent transcriptomics studies demonstrated that brain mural cells are enriched in vitronectin mRNA and protein immunoreactivity (Campbell et al., 2017). In both the brain and leptomeninges of LepRb-Cre^tdTomato^ mice, the abluminal surface of the vast majority of tdTomato-positive perivascular cells was decorated by with vitronectin immunoreactivity (**Figures 5B,C**). Platelet derived growth factor receptor beta (PDGFRB) has commonly been used in the past to identify pericytes in the mouse brain (Winkler et al., 2010). Strikingly, PDGFRB immunoreactivity was present at the surface of most tdTomato-tagged perivascular cells in both the meninges and around brain blood vessels (**Figures 6A,B**).

### Perivascular Cells Genuinely Express the Leptin Receptor

**FIGURE 7:**
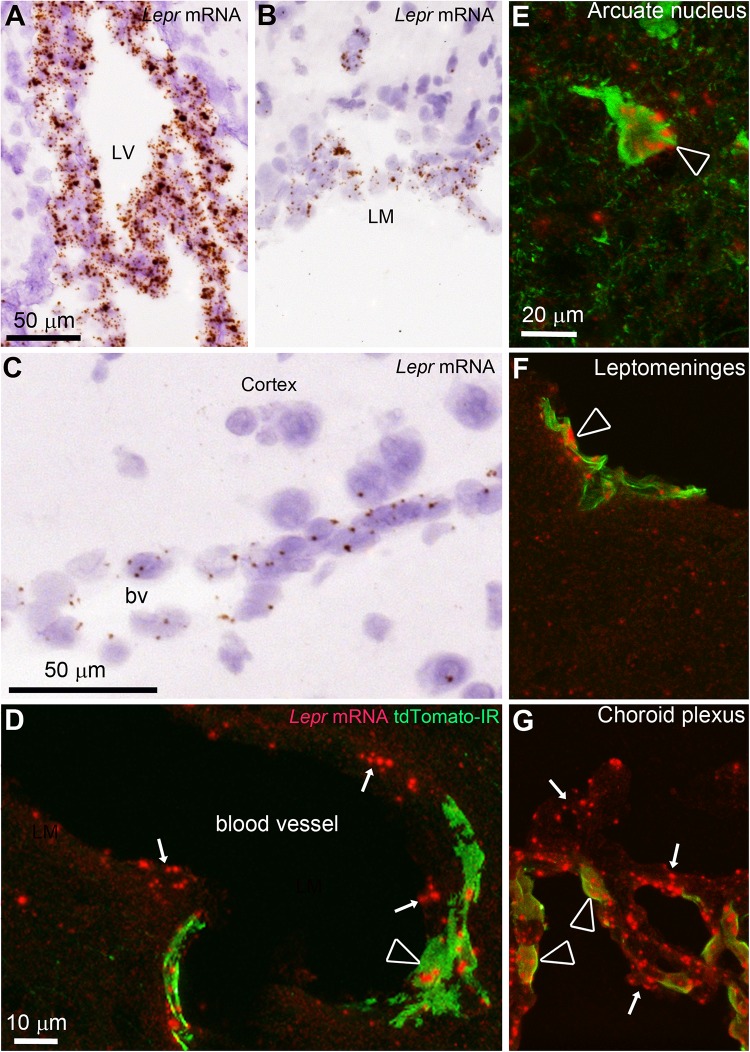
*In situ* hybridization (ISH) for the leptin receptor (all isoforms) in the mouse brain. **(A–C)** ISH signals (brown) were detected in the choroid plexus, leptomeninges and blood vessels of C57Bl/6J mice. Tissue was counterstained with H&E. **(D)** In LepRb-Cre-tdTomato mice, ISH signals (red) were detected in both tdTomato-negative and -positive cells making up blood vessels (arrows) including endothelial and perivascular cells (arrowhead), respectively. **(E)** Hypothalamic neurons positive for tdTomato almost always coexpressed *Lepr* mRNA (arrowhead). **(F,G)** Td-tomato-labeled meningeal cells and choroid plexus cells also coexpressed *Lepr* mRNA. However, not all LepR mRNA-expressing epithelial cells in the choroid plexus were tdTomato-labeled cells (arrows). Scale bar in **(A)** applies to **(B)**. Scale bar in **(E)** applies through **(G)**.

To our knowledge, the leptin receptor mRNA has not been described before in intracranial perivascular cells. Here, we sought to confirm that the leptin receptor mRNA was expressed in tdTomato-labeled cells using chromogenic *in situ* hybridization techniques. A probe recognizing all the leptin receptor isoforms was validated on brain sections of adult C57Bl/6J mice using DAB as a chromogen (**Supplementary Figures S2A–C**). DAB accumulated in leptin receptor-expressing cells of the mouse brain including hypothalamic nuclei, following a distribution that conformed well to that of tdTomato-positive cells. Positive and negative control experiments confirmed the specificity of the signals (**Supplementary Figure S2**). Within non-neuronal structures, *Lepr* mRNA expression was evident in the entire choroid plexus, the leptomeninges and the mouse brain vasculature (**Figures 7A–C**). Double-labeling for *Lepr* mRNA using FastRed as a chromogen, combined with immunohistochemistry for the tdTomato protein, was performed on brain sections from LepRb-Cre^tdTomato^ mice (**Figures 7D–G**). We found that the vast majority of tdTomato-tagged cells contained signals for the *Lepr* mRNA including perivascular cells (>79%), hypothalamic neurons (>90%), and choroid plexus cells (>91%) (**Figures 7D–G**). In contrast, tdTomato-tagged neurons in the hippocampus, globus pallidus, and cortex, expressed little LepR mRNA (<7%). This is explained by the fact that neurons located in these forebrain structures may only transiently express LepRb during development (Caron et al., 2010). Intriguingly, *in situ* hybridization signals were also detected in tdTomato-negative structures which presumably solely expressed the short-isoforms of the leptin receptor. These structures included many cells in the choroid plexus epithelium and the lining of isolated blood vessels (**Figures 7D,G**). In particular, *Lepr* mRNA was also observed in tdTomato-negative cells immediately adjacent or beneath tdTomato-tagged perivascular cells, which likely correspond to endothelial cells (**Figure 7D**). Of note, double chromogenic *in situ* hybridization further confirmed that the *Lepr* mRNA extensively colocalized with the transcript for *Pdgfrb* (**Supplementary Figures S3A,B**), but not that of Gfap or Aif1 (**Supplementary Figures S3C–F**).

### Altered Perivascular Markers Expression in LepRb Deficient Mice

**FIGURE 8:**
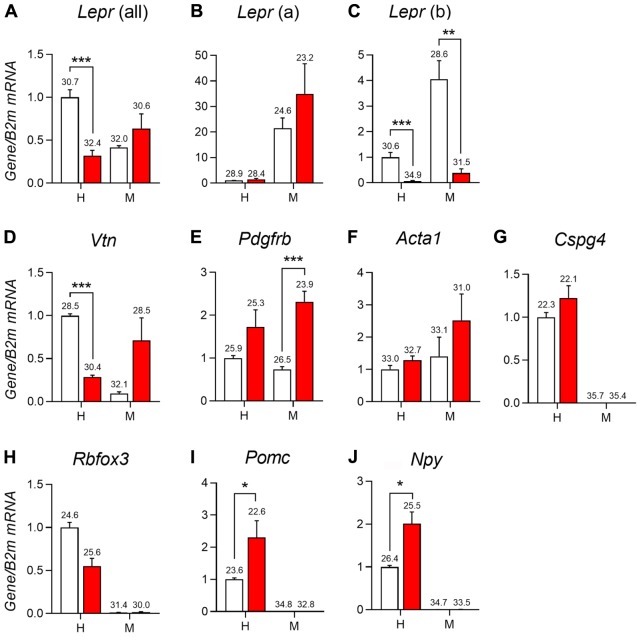
qPCR analysis of the mediobasal hypothalamus (H) and dura mater meninges (M) of LepRb-deficient mice (red) and control littermates (white). **(A–C)** Expression of different isoforms of the leptin receptors including Lepr mRNA (all isoform), Lepr(a) (short-isoform), and Lepr(b) (long isoform) in the hypothalamus and meninges. **(D–G)** Expression of select markers of mural cells in the hypothalamus and meninges including vitronectin (Vtn), Platelet-derived growth factor receptor beta (Pdgfrb), Alpha-actin-1 (Acta1), and Chondroitin Sulfate Proteoglycan (Cspg4). **(H–J)** Expression of different neuronal genes in the hypothalamus and meninges including RNA Binding Protein Fox-1 Homolog 3 (Rbfox3), Pomc (Pro-opiomelanocortin), and Neuropeptide Y (Npy). The value above each bar graph is the cycle number at threshold. Asterisks identify results that were significantly different from chow at *p* < 0.05, *p* < 0.01, and *p* < 0.001.

Recent studies have associated leptin and LepRb deficiency with pericytopathy in peripheral organs including the liver, pancreas, and bone marrow (Choi et al., 2010; Chien et al., 2016; Yue et al., 2016; Haley and Lawrence, 2017; Xie et al., 2017). Using qPCR studies, we further characterized the expression of different leptin receptor isoforms and perivascular markers in the hypothalamus and meninges of LepRb-deficient mice. As anticipated, the hypothalamus predominantly expressed LepRb (**Figures 8A–C**). In mice lacking functional LepRb (Berglund et al., 2012), the hypothalamic expression for *Lepr* (all isoforms) and *Leprb* was significantly reduced, but *Lepra* hypothalamic expression was not (**Figures 8A–C**). In the dura matter, *Lepra* was expressed at much higher levels than LepRb (**Figures 8A–C**). Nonetheless, LepRb mRNA was detectable and its levels were reduced in the LepRb-deficient mice, thus confirming the presence of a sizable population of LepRb-expressing cells (**Figure 8C**). We also assessed the expression of the perivascular markers vitronectin, Pdgfbr, Acta1 (α-SMA), and Cspg4 (NG2) (Winkler et al., 2011; Hill et al., 2015; Attwell et al., 2016; Trost et al., 2016). In the hypothalamus of LepRb-deficient mice, vitronectin expression was significantly lower (**Figure 8D**). Other perivascular markers were unchanged (**Figures 8E–G**). In the dura matter, we observed a trend toward increased expression of vitronectin in LepRb-deficient mice (**Figure 8D**). Meningeal Pdgfrb expression was also significantly increased in LepRb-deficient mice (**Figure 8E**). The expression of *Acta1* and *Cspg4* remained unchanged in both genotypes, and *Cspg4* was not detectable in meninges (**Figures 8F,G**). Control neuronal genes included Rbfox3, Pomc and Npy (**Figures 8H–J**). As expected, these genes were only expressed in the hypothalamus and were regulated upon LepRb-deficiency (Mizuno et al., 1998).

**FIGURE 9:**
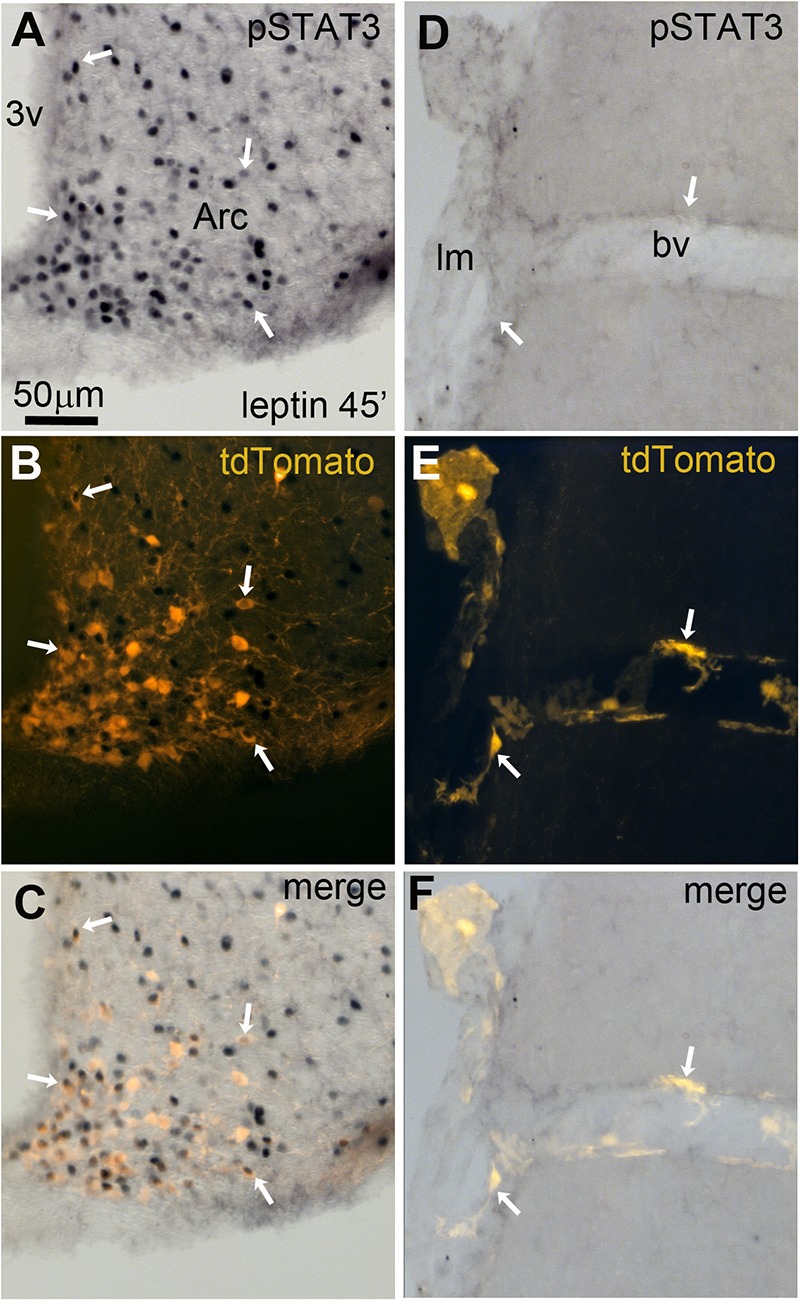
Leptin-induced pSTAT3 in the brain of LepRb-Cre-tdTomato mice. **(A–C)** In the arcuate nucleus, nuclear immunoreactivity for pSTAT3 was seen in a large number of tdTomato-labeled neurons (arrows). **(D–F)** No detectable pSTAT3 immunoreactivity was present within the leptomeninges and perivascular space. Scale bar in **(A)** applies through **(F)**. 3v, third ventricle; Arc, arcuate nucleus of the hypothalamus; bv, blood vessel; lm, leptomeninge.

As previously discussed, many leptin-related cellular effects on neurons have been linked to the recruitment of pSTAT3 (Fernandes et al., 2015). In peripheral pericytes, pSTAT3 may also play a key role in mediating leptin’s actions (Yue et al., 2016; Riu et al., 2017). Thus, we assessed the ability of exogenous leptin to stimulate pSTAT3 in tdTomato-tagged cells. Forty-five minutes after the intraperitoneal injection of leptin in LepRb-Cre^tdTomato^ mice, pSTAT3 immunoreactivity accumulated in the nucleus of many tdTomato-tagged neurons of the arcuate nucleus (**Figures 9A–C**). In contrast, we never observed any pSTAT3 immunoreactivity in the meningeal or perivascular space (**Figures 9D–F**).

## Conclusion

This study demonstrated that the only intracranial non-neuronal cells that express the leptin receptors (including LepRb) are cells making up the blood-brain barrier, including subsets of meningeal perivascular cells, endothelial cells and choroid plexus cells. Moreover, LepRb-deficiency was found to be associated with altered expression for several markers of pericytes, suggesting the presence of pericytopathy. As discussed below, we suggest that leptin signaling in perivascular cells may play a role in the integrity of the intracranial perivascular space and, consequently, provide a link between obesity and numerous brain diseases.

### Leptin Signaling in Non-neuronal Brain Cells

Past studies described the expression of LepRa in non-neuronal brain cells, including most notably meningeal, endothelial and choroid plexus cells (Bjorbaek et al., 1998; Elmquist et al., 1998; Carlo et al., 2007; Pan et al., 2012). Our anatomical and qPCR data largely agree with and extend these observations by showing LepRa expression in cells making up the blood brain barrier and meninges. Emerging literature also suggests that functional LepRb signaling occurs in astrocytes, tanycytes, ependymal cells, and microglia cells (Cottrell et al., 2009; Hsuchou et al., 2009b; Balland et al., 2014; Kim et al., 2014). In contrast, other studies, including single-cell transcriptomics studies, have failed to detect enrichment of leptin receptor mRNA in hypothalamic or extra-hypothalamic glial cells (Allison et al., 2015; Campbell et al., 2017). Our data agree with the view that glial cells in the normal mouse brain, including astrocytes and microglia, do not express LepRb. Although one can reasonably be confident that glial cells in the adult brain do not express leptin receptors, it cannot be ruled out that glial cells could produce LepRb under pathological conditions. Moreover, it is notable that recent RNAseq analysis indicate that GFAP is not a universal marker for astrocytes and that, consequently, we may have missed Lepr-expressing astrocytes with low/near-undetectable GFAP (Zeisel et al., 2015). Leptin may also indirectly influence glial cells activities (Djogo et al., 2016). It remains puzzling that, if leptin receptors are absent from astrocytes, the deletion of LepRb in GFAP-producing cells resulted in a metabolic phenotype (Jayaram et al., 2013; Kim et al., 2014; Rottkamp et al., 2015). However, it must be stressed that the deletion of LepRb in peripheral GFAP cells may contribute to the metabolic actions of leptin. Indeed, GFAP is produced in many peripheral cells, including hepatic perivascular cells known as stellate cells (Yang et al., 2008), pancreatic perivascular cells (Omary et al., 2007), Schwann cells (Triolo et al., 2006), and enteric astrocytes (Martin et al., 2012; McClain et al., 2015). Among the aforementioned cell types, hepatic GFAP-stellate cells are known to express LepRb (Saxena et al., 2002; Choi et al., 2010). Future studies are therefore warranted to establish the role of LepRb in peripheral GFAP cells.

### Categorization of Perivascular Cells

Based on our data, we are confident that at least some subsets of intracranial pericytes expressed *Leprb* and *Lepra*. We are not aware of published data on leptin signaling in intracranial perivascular cells. In fact, RNA-Seq experiments have failed to detect LepR in cortical pericytes (Zeisel et al., 2015), probably due to the fact that Lepr is mostly enriched in meningeal pericytes. In the periphery, however, several studies have identified LepRb-Cre activity or LepRb mRNA in perivascular cells of the bone marrow, intestines, and liver (Saxena et al., 2004; Rajala et al., 2014; Zhou et al., 2014), thereby reinforcing the validity of our own findings. We also observed tdTomato-positive pericytes in peripheral tissues including the intestines (not shown). In our study, tdTomato-positive pericytes were preferentially localized in the meninges rather than the brain parenchyma, suggesting the existence of different subtypes of intracranial pericytes that either do or do not express LepRb. It must be stressed that pericytes are highly heterogeneous cells sharing overlapping features with other cell types including smooth muscle cells (Winkler et al., 2011; Attwell et al., 2016). This has rendered the categorization of pericytes a debated area of research. Despite the lack of consensus on how to identify and classify pericytes, vitronectin has recently been identified as a molecular marker for cerebral mural cells, including pericytes (He et al., 2016). In our hands, vitronectin and PDGFBR immunoreactivities marked the surface of most tdTomato-tagged cells. Pdgfrb and LepR mRNAs were also colocalized in the leptomeninges. Moreover, the morphology of most tdTomato-tagged cells was undeniably reminiscent of that described in the literature for pericytes based on varied antibody and reporter models (Winkler et al., 2011; Attwell et al., 2016). Lastly, markers for pericytes were influenced by leptin deficiency. Thus, we are confident that most non-neuronal tdTomato-tagged cells in the meninges were pericytes and that LepRb-Cre mice are a useful tool for visualizing subsets of pericytes. Nonetheless, additional work using electron microscopy and double immunostaining is needed to further narrow down the subtype(s) of LepRb-expressing mural cells.

#### Potential Roles of Leptin Signaling in Intracranial Pericytes

Intracranial pericytes have been linked to numerous functions, including the regulation of blood flow, neoangiogenesis, brain repair, inflammatory responses, and the transmigration of immune cells (Winkler et al., 2011; Trost et al., 2016). However, the physiological role of leptin signaling in cerebral pericytes remains open to speculation. In our animals, tdTomato-positive pericytes were mainly enriched in the short isoforms of the leptin receptor. Interestingly, mice selectively lacking LepRa have reduced transport of endogenous leptin across the blood-brain barrier (Kastin et al., 1999). Hence, it is conceivable that in pericytes, as well as in endothelial and choroid plexus cells, LepRa may be involved in the intracerebral transport of leptin. Evidence of leptin modulatory actions on neuroinflammation and leukocyte infiltration into the brain also exists (Rummel et al., 2008; Aguilar-Valles et al., 2014). In particular, the latter studies proposed that leptin signaling in undetermined meningeal cells could facilitate the movement of immune cells across the blood-brain barrier. Furthermore, leptin has been reported to be neuroprotective in numerous brain diseases (Signore et al., 2008; Davis et al., 2014). It is therefore tempting to speculate that leptin signaling may also play a role in brain diseases with an inflammatory component, perhaps via the regulation of the perivascular coverage and leukocyte infiltration. Lastly, several studies reported peripheral pericyte loss in mice deficient for leptin or LepRb (Chien et al., 2016; Haley and Lawrence, 2017; Xie et al., 2017), and further demonstrated that LepRb signaling played an important role in the proliferation and differentiation of peripheral pericytes (Saxena et al., 2002; Choi et al., 2010; Zhou et al., 2014; Yue et al., 2016). This suggests that intact LepRb signaling is required for normal perivascular cells activities. Here, we also found altered vitronectin and PDGFBR expression in the meninges and hypothalamus of LepRb-deficient mice. Ultimately, reduced pericytic coverage in LepRb-deficient mice may explain the exaggerated brain damage observed following experimental trauma in ob/ob and db/db mice (McColl et al., 2010; Tureyen et al., 2011). Additional studies are therefore critical to assess the influence of leptin signaling on cerebral pericyte genesis in health and disease.

As a final remark, we found that leptin failed to induce pSTAT3 in brain perivascular cells. *In vivo*, it may be that competitive binding to the highly expressed short-isoforms of the leptin receptor prevented exogenous leptin from stimulating pSTAT3 in perivascular cells. It could also be that leptin-associated pSTAT3 may be more transient in pericytes than in neurons, as is the case in tanycytes (Balland et al., 2014). Alternatively, signaling pathways other than the JAK-STAT pathway may play a greater role in pericytes. For instance, studies have shown that leptin predominantly recruits the Akt and Erk pathways rather than pSTAT3 in hepatic perivascular cells (Lang et al., 2004; Saxena et al., 2004). In conclusion, more studies are needed to determine the exact intracellular pathways underlying leptin’s actions on intracranial pericytes.

## Author Contributions

XY performed histology; AC performed qPCR; HW provided expertise; LG performed microscopy, wrote the manuscript and designed most of the experiments.

## Conflict of Interest Statement

The authors declare that the research was conducted in the absence of any commercial or financial relationships that could be construed as a potential conflict of interest.

## Supplementary Material

FIGURE S1 Representative images of tdTomato fluorescence in whole mounts of the dura mater. We used 15 unsexed LepRb-Cre-tdTomato neonates on postnatal days 0 (P0), 3(P3), 6 (P6), and 15 (P15) (*n* = 3–4 per time point). Mice were perfused with formalin as described before and their meninges were immediately removed. The dura mater was rinsed in PBS and placed as flat as possible on a histology slides. We next added a few drop of Vectashield and placed a coverslip on the tissue. Observations were repeated in a total of 3 mice per postnatal stage.

FIGURE S2 Leptin receptors-expressing cells were studied using a novel chromogenic ISH technique (RNAScope^®^). Control experiments were performed in the brain of 3 C57/Bl6 male mice. **(A)** As anticipated, the ISH signal (brown precipitate) for LepR mRNA was specifically distributed in brain sites well-known to express leptin receptors including the arcuate nucleus (Arc) and ventromedial hypothalamus (VMH). **(B)** As a positive control, we detected the expression of Ppib mRNA across the entire mouse brain including neurons and non-neuronal cells. **(C)** As a negative control, we performed ISH using a probe recognizing the prokaryotic gene dapB. Brain sections were completely devoid of a signal, therefore demonstrating that our approach generated virtually no unspecific background. 3v, third ventricle.

FIGURE S3 Double chromogenic in situ hybridization for *Lepr* and select non-neuronal markers. Briefly, brain sections from two young C57/Bl6 males were prepared as described for chromogenic *in situ* in the main manuscript. The Pretreatment step consisted of Retrieval 2 followed by Protease Plus. For detecting hybridization signals, we used the Duplex kit (322500 RNAscope^®^ 2.5 HD Duplex Detection Reagents) following the manufacturer’s instructions. The probes are indicated in **Table 1**. Signals for the *Lepr* mRNA were detected as blue dots, while the other genes (*Gfap*, or *Pdgfrb*, or *Aif1*) were detected as red dots. Of note, the tissue was lightly counterstained with hematoxylin. **(A,B)** Pdgfrb and LepR signals coincided extensively in the leptomeninges and isolated cortical blood vessels (black arrows). Nonetheless, a majority of Pdgfb-positive cells remained negative for the leptin receptor. **(C,D)** Gfap was prominent in the glia limitans and around blood vessels. However, Gfap and LepR signals were not seen in the same profiles. We made similar observations in the hypothalamus (not shown). **(E,F)** Aif1 mRNA was distributed in microglial cells across the cortex and never colocalized with *Lepr*. Overall, our double labeling supplementary data are in agreement with the observations made in the main manuscript using the LepR-Cre mouse, and further confirm that *Lepr* mRNA is expressed in meningeal pericytes, to a lesser extent in parenchymal pericytes, but never in macroglial cells.
