# Supplementary figures and images for: Leptin Receptor Expression in Mouse Intracranial Perivascular Cells
Source: Front Neuroanat. 2018 Jan 23;12:4. doi: 10.3389/fnana.2018.00004 (PMC5787097; doi:10.3389/fnana.2018.00004)

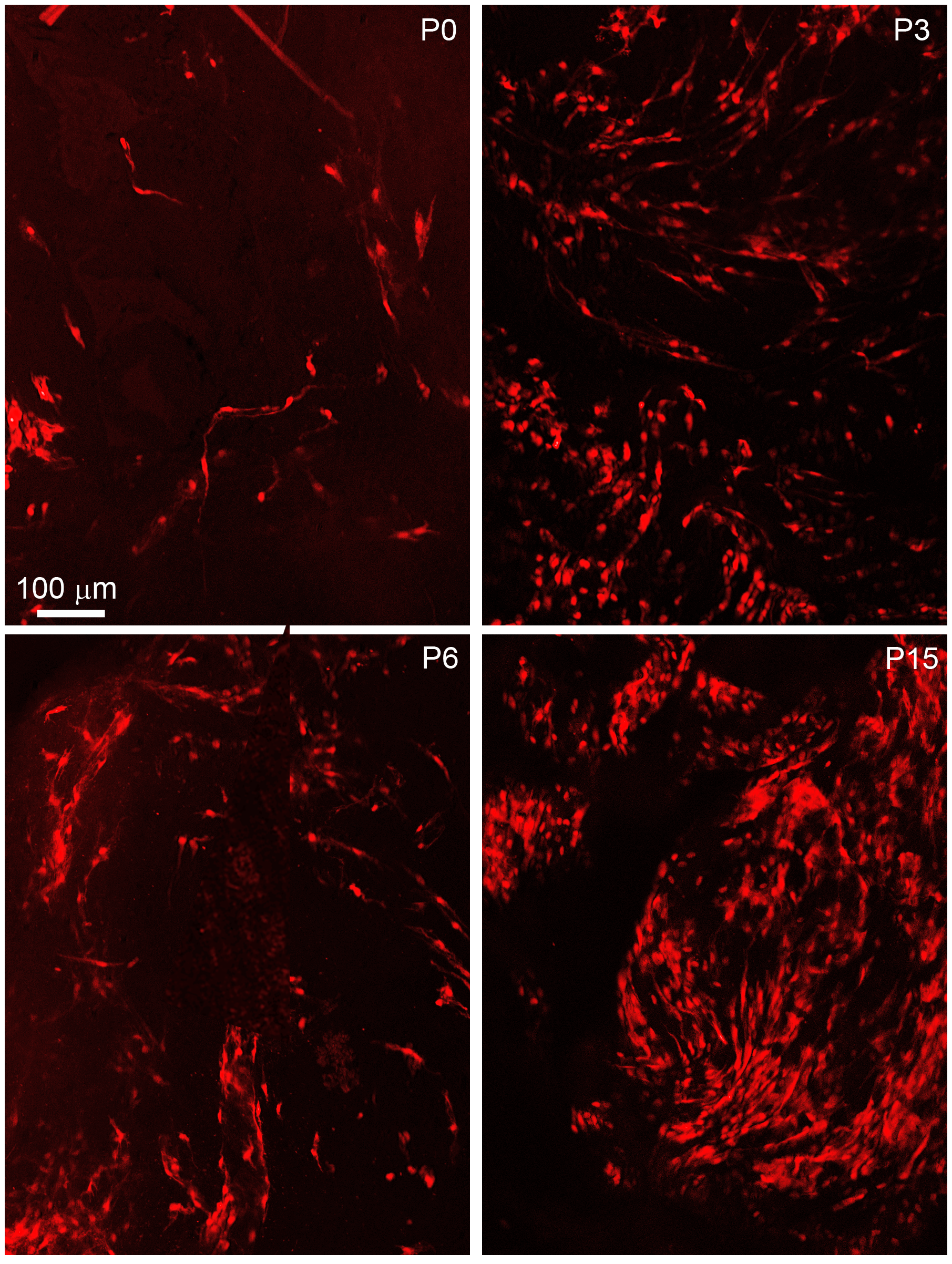

Supplement: FIGURE S1 — Representative images of tdTomato fluorescence in whole mounts of the dura mater. We used 15 unsexed LepRb-Cre-tdTomato neonates on postnatal days 0 (P0), 3(P3), 6 (P6), and 15 (P15) (n = 3–4 per time point). Mice were perfused with formalin as described before and their meninges were immediately removed. The dura mater was rinsed in PBS and placed as flat as possible on a histology slides. We next added a few drop of Vectashield and placed a coverslip on the tissue. Observations were repeated in a total of 3 mice per postnatal stage. [file Image_1.TIF]

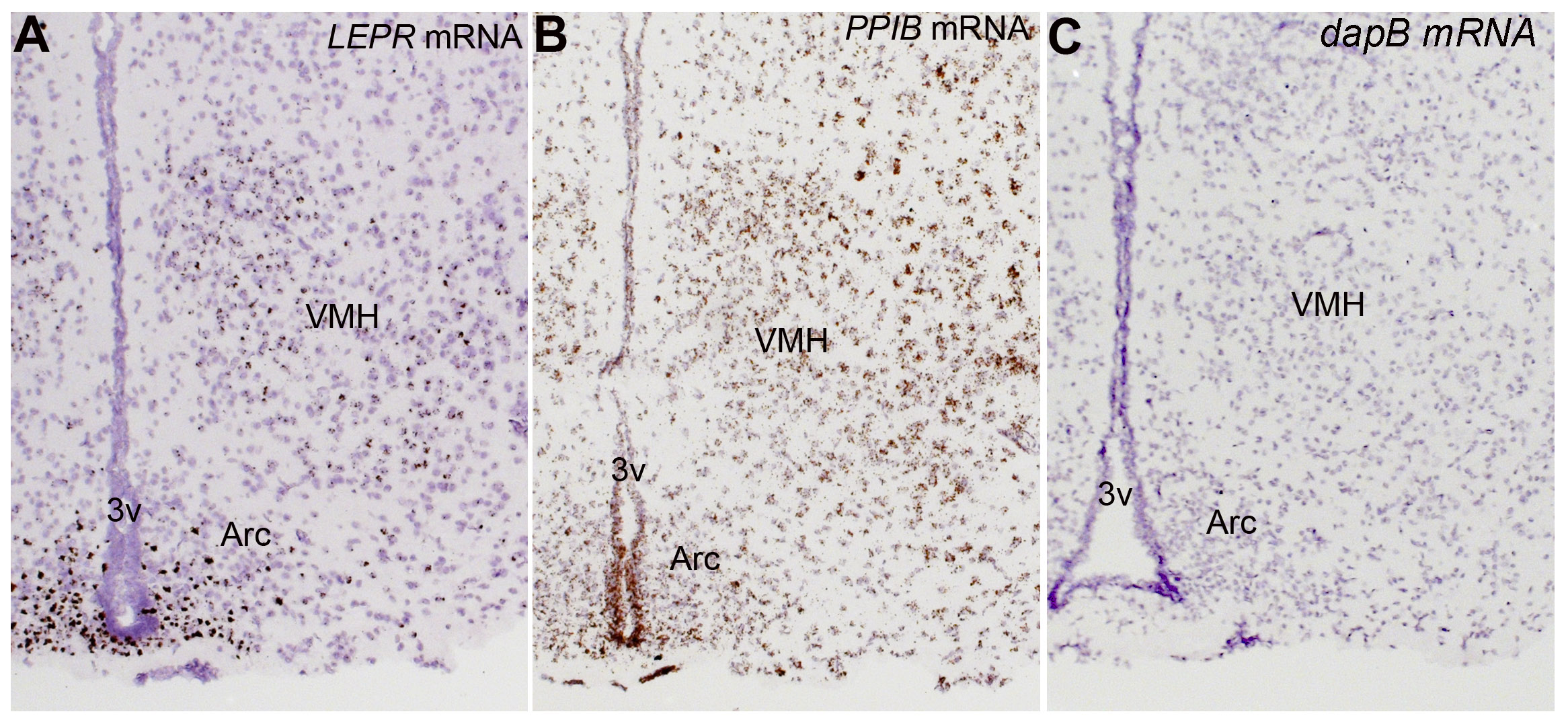

Supplement: FIGURE S2 — Leptin receptors-expressing cells were studied using a novel chromogenic ISH technique (RNAScope®). Control experiments were performed in the brain of 3 C57/Bl6 male mice. (A) As anticipated, the ISH signal (brown precipitate) for LepR mRNA was specifically distributed in brain sites well-known to express leptin receptors including the arcuate nucleus (Arc) and ventromedial hypothalamus (VMH). (B) As a positive control, we detected the expression of Ppib mRNA across the entire mouse brain including neurons and non-neuronal cells. (C) As a negative control, we performed ISH using a probe recognizing the prokaryotic gene dapB. Brain sections were completely devoid of a signal, therefore demonstrating that our approach generated virtually no unspecific background. 3v, third ventricle. [file Image_2.JPEG]

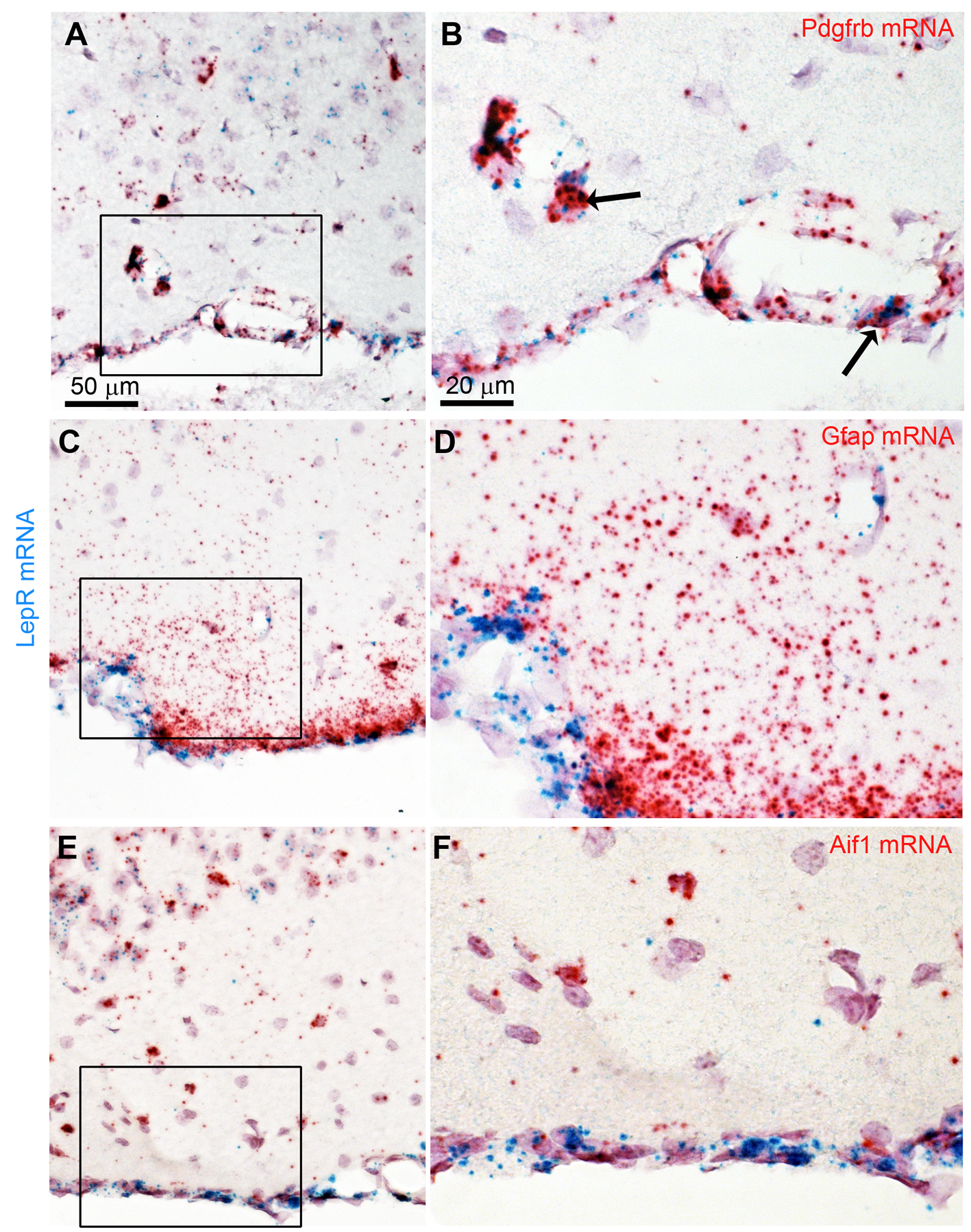

Supplement: FIGURE S3 — Double chromogenic in situ hybridization for Lepr and select non-neuronal markers. Briefly, brain sections from two young C57/Bl6 males were prepared as described for chromogenic in situ in the main manuscript. The Pretreatment step consisted of Retrieval 2 followed by Protease Plus. For detecting hybridization signals, we used the Duplex kit (322500 RNAscope® 2.5 HD Duplex Detection Reagents) following the manufacturer’s instructions. The probes are indicated in Table 1. Signals for the Lepr mRNA were detected as blue dots, while the other genes (Gfap, or Pdgfrb, or Aif1) were detected as red dots. Of note, the tissue was lightly counterstained with hematoxylin. (A,B) Pdgfrb and LepR signals coincided extensively in the leptomeninges and isolated cortical blood vessels (black arrows). Nonetheless, a majority of Pdgfb-positive cells remained negative for the leptin receptor. (C,D) Gfap was prominent in the glia limitans and around blood vessels. However, Gfap and LepR signals were not seen in the same profiles. We made similar observations in the hypothalamus (not shown). (E,F) Aif1 mRNA was distributed in microglial cells across the cortex and never colocalized with Lepr. Overall, our double labeling supplementary data are in agreement with the observations made in the main manuscript using the LepR-Cre mouse, and further confirm that Lepr mRNA is expressed in meningeal pericytes, to a lesser extent in parenchymal pericytes, but never in macroglial cells. [file Image_3.TIF]
